# Precision Oncology in Canada: Converting Vision to Reality with Lessons from International Programs

**DOI:** 10.3390/curroncol29100572

**Published:** 2022-09-30

**Authors:** Geoffrey Liu, Winson Y. Cheung, Harriet Feilotter, Jackie Manthorne, Tracy Stockley, ManTek Yeung, Daniel J. Renouf

**Affiliations:** 1 Princess Margaret Cancer Centre, University Health Network, 610 University Ave, Toronto, ON M5G 2M9, Canada; 2 Dalla Lana School of Public Health, University of Toronto, 155 College St, Toronto, ON M5T 3M7, Canada; 3 Department of Oncology and Tom Baker Cancer Centre, Cumming School of Medicine, University of Calgary, 1331 29th Street NW, Calgary, AB T2N 4N2, Canada; 4 Health Services Research Program, Cancer Control Alberta, Alberta Health Services, 10030—107 Street NW, Edmonton, AB T5J 3E4, Canada; 5 Ontario Institute for Cancer Research, 661 University Ave, Toronto, ON M5G 0A3, Canada; 6 Department of Pathology and Molecular Medicine, Queen’s University, 88 Stuart Street, Kingston, ON K7L 3N6, Canada; 7 Canadian Cancer Survivor Network, 1750 Courtwood Crescent, Ottawa, ON K2C 2B5, Canada; 8 Division of Clinical Laboratory Genetics, Laboratory Medicine Program, University Health Network, 200 Elizabeth Street, Toronto, ON M5G 2C4, Canada; 9 Department of Laboratory Medicine and Pathobiology, University of Toronto, 1 King’s College Circle, Toronto, ON M5S 1A8, Canada; 10 Hoffmann-La Roche Limited, 7070 Mississauga Road, Mississauga, ON L5N 5M8, Canada; 11 Department of Medicine, University of British Columbia, 2775 Laurel Street, Vancouver, BC V5Z 1M9, Canada; 12 BC Cancer, 600 West 10th Avenue, Vancouver, BC V5Z 4E6, Canada

**Keywords:** precision oncology, personalized medicine, precision medicine, clinical utility, real-world evidence, next-generation sequencing, tumour-agnostic, funding

## Abstract

Canada’s healthcare system, like others worldwide, is immersed in a process of evolution, attempting to adapt conventional frameworks of health technology assessment (HTA) and funding models to a new landscape of precision medicine in oncology. In particular, the need for real-world evidence in Canada is not matched by the necessary infrastructure and technologies required to integrate genomic and clinical data. Since healthcare systems in many developed nations face similar challenges, we adopted a solutions-based approach and conducted a search of worldwide programs in personalized medicine, with an emphasis on precision oncology. This search strategy included review articles published between 1 January 2016 and 1 March 2021 and hand-searches of their reference lists for relevant publications back to 1 December 2005. Thirty-nine initiatives across 37 countries in Europe, Australasia, Africa, and the Americas had the potential to lead to real-world data (RWD) on the clinical utility of oncology biomarkers. We highlight four initiatives with helpful lessons for Canada: Genomic Medicine France 2025, UNICANCER, the German Medical Informatics Initiative, and CANCER-ID. Among the 35 other programs evaluated, the main themes included the need for collaboration and systems to support data harmonization across multiple jurisdictions. In order to generate RWD in precision oncology that will prove acceptable to HTA bodies, Canada must take a national approach to biomarker strategy and unite all stakeholders at the highest level to overcome jurisdictional and technological barriers.

## 1. Introduction

### 1.1. Precision Oncology: The Vision and Reality

In its 2020 statement, the International Consortium for Personalised Medicine (ICPerMed) concluded that, “(Precision Medicine) is not so much a paradigm change as the evolution of medicine in a biotechnology and data-rich era” [1]. The convergence of three platforms—digital health, data science, and precision therapies—are driving the transformation [2]. Canada’s healthcare system, in common with others worldwide, is in this process of evolution, attempting to adapt conventional frameworks of medical practice, health technology assessment (HTA), and funding models to the new landscape [3].

The potential benefits of precision medicine are now clear. These include: improved diagnosis and monitoring; therapies that target a patient’s individual genomic profile or that of a target tumour; reduced patient exposure to ineffective treatments; and, cost savings from better targeting of expensive therapies to those likeliest to benefit [2,4]. Comprehensive molecular profiling of a disease has accelerated research and clinical development of new treatments; however, it is also becoming evident that the complexity of the changes needed to deliver precision medicine in a public healthcare system requires a ‘precision medicine ecosystem’ comprising government, industry, professional societies, healthcare providers, researchers, data scientists, and payers [2,5]. Without these changes, public access to innovative technologies lags significantly behind their discovery. Nowhere is this more true than in oncology.

In the past decade, molecular profiling of oncology biomarkers has reorganized the number of subtypes of cancer into a new taxonomy [2] based on the pathways that drive tumourigenesis, rather than histology or clinical history alone. This reorganization includes the possibility of identifying tumour-agnostic agents targeting the specific molecular dysfunction that promotes uncontrolled cell proliferation in a given patient’s cancer [2,4,6,7,8]. The new molecular taxonomy of cancer requires clinical researchers to employ novel clinical trial designs such as ‘basket’ and ‘umbrella’ trials, as well as adaptive trials that alter treatment based on real-time tumour response [9,10,11,12]. These novel study designs have led to accelerated regulatory approvals of several targeting agents without the typical randomized phase 3 studies, and yet public reimbursement continues to lag. In a recent report, Canada ranks 19th out of 20 in Organisation for Economic Co-operation and Development (OECD) countries for the number of days between regulatory approval of a new drug and its public reimbursement listing [13,14]. 

While these innovative approaches provide high-quality safety and efficacy data on investigational molecules, the basket and umbrella study designs pose new challenges for HTA processes that require robust comparative data to assess costs and benefits. Uncertainty in economic analyses increases when the genomic alteration under investigation is exceptionally rare, and when the underlying data originate from multiple tumour sites with widely differing natural histories of disease. As early as 2011, the OECD called for regulatory and reimbursement procedures to be adapted to the specificities of novel biomarker-based clinical tests and harmonized across jurisdictions [15]. Only recently has any such adaptation begun in regulatory approval and funding of comprehensive biomarker testing. Progress in this regard is not uniform across Canadian jurisdictions.

In Canada, more than 20 targeted therapies approved by Health Canada now require testing for molecular biomarkers, and three recent therapies—pembrolizumab, larotrectinib, and entrectinib—were approved based on basket trial data [16]. To date, only larotrectinib has received a positive recommendation for public funding. Even in this case, funding recommendations differ between Quebec and the rest of Canada, reflecting divergent views of different regulatory authorities regarding tumour-agnostic use of this product.

### 1.2. The Canadian Situation

Canada and other countries face multiple barriers to wide adoption of targeted cancer therapies. HTA frameworks have not readily adapted to accommodate opportunities for funding based on molecular biomarker-directed therapies given regulatory approval from non-randomized clinical trial data. In most provinces, reimbursement for molecularly matched medicines has been uncertain, with the exception of a limited suite of targeted therapies (e.g., imatinib [17], erlotinib [18], brigatinib [19] and crizotinib [20]) for which randomized studies were feasible. Furthermore, drugs such as entrectinib and crizotinib have multiple targets, and the current paradigm of HTA assessments has mostly focussed on evaluating one marker at a time. 

Canadian healthcare is decentralized: provinces and territories fund, regulate, and provide health services. Although health technologies such as drugs and proprietary tests are reviewed federally by Health Canada, final reimbursement for them is provided by each healthcare jurisdiction. Centralized HTA analyses for clinical effectiveness, cost-effectiveness, and implementation challenges may be carried out by the Canadian Agency for Drugs and Technologies in Health (CADTH) and in Quebec, the Institut National d’Excellence en Santé et en Services Sociaux. Oncology drugs are assessed by the CADTH pan-Canadian Oncology Drug Review. However, no parallel organization monitors laboratory investigations into the biomarkers associated with targeted therapies.

Precision oncology informed by basket and umbrella studies does not easily conform to the existing HTA processes in Canada. Some provincial organizations, such as Génome Québec [21], have commenced development of HTA assessment structures to fill the gap, although none are yet in use.

While Canadian HTA authorities’ thinking may be shifting, the following case, based on a previous HTA evaluation, illustrates some challenges that tumour-agnostic therapies continue to face [22]: the developers of a hypothetical Drug A submitted an application to an HTA assessment body for reimbursement 3 months prior to the anticipated date of regulatory approval of the product. The company requested coverage of the drug for patients carrying the target mutation in tumours of any type; no other therapies were available that targeted this rare mutation. To support its application, the company provided data from a pooled analysis of four basket trials involving 924 patients with the gene variant, in eight different tumour types. The pooled analysis showed an overall response rate (ORR) of 92%. Progression-free survival was 25 months, but the overall survival (OS) benefit could not be determined.

The HTA assessment body initially decided not to recommend public reimbursement of Drug A. While acknowledging that Drug A was well tolerated, the committee had difficulty concluding the drug had a net benefit. In justifying its negative decision, the group noted the open-label design of the basket trials, the absence of a standard-of-care comparator, and the small number of patients for each tumour type. They also mentioned the heterogeneity of the patient populations and study designs, as well as the lack of survival data specific to patients carrying the target variant. ORR was not accepted as a surrogate for OS, and quality of life data provided were not sufficiently compelling to change the decision. At the time, the HTA body determined that the limitations of the pooled data and the lack of clarity on the clinical significance of the mutation left the cost-effectiveness of the therapy uncertain.

Thus, novel medicines such as Drug A face the attendant challenges of treating any rare disease: small patient populations, no acceptable comparator, heterogeneous phenotypes, and a dearth of information on natural history. These barriers to funding cannot be overcome solely by better clinical trials; real-world data (RWD) may help overcome these issues.

### 1.3. HTA Assessment Challenges Specific to Next-Generation Sequencing

When deliberating on molecularly matched drug funding requests, HTA bodies must also assess the economic impact of the companion testing required to identify patients. As with umbrella and basket trial outcome data, the lack of robust comparative data for next-generation sequencing (NGS)-based studies complicates assessment of this technology’s health economic impact. The complexity of assessing the economic impact of NGS cost increases when many patients need to be tested to identify a rare genomic alteration or when multiple tumour sites with varying rates of testing need to be incorporated into new testing algorithms. To overcome this complexity, professional organizations and commentators in many countries have offered various recommendations on access to NGS for precision oncology [6,7,11,16,23,24]. In Canada, the decision largely rests on budgetary considerations: individual laboratories decide how best to measure a biomarker based on the funds available to them following approval and funding of that biomarker for clinical use. This is an evolving process in Canada, where regulators have recently presented themselves as open to incorporating the incremental cost of NGS testing when assessing drug cost-effectiveness [16].

The utility of testing many genes simultaneously is clear, allowing for the rapid comprehensive molecular characterization of a tumour while preserving precious tissue. For example, Perdrizet et al. compared standard-of-care testing in Ontario (oncogenic mutations in *EGFR*, and rearrangements in *ALK* and *ROS1*) to a broadened 15-gene NGS panel in 433 non-squamous non-small cell lung cancer (NSCLC) specimens [25,26]. For a modest incremental cost relative to single-gene analysis, they were able to identify actionable targets distinct from *EGFR* and *ALK* in 3.2% of specimens. Other authors have commented that, “in a near future, testing with a 300-gene panel may have a similar cost to that of testing 5 or 6 alterations individually” [27,28].

On the downside, the cost of providing genomic testing broadly to all cancer patients represents a large health resource investment, with uncertain benefit. Notably, when assessing the impact of NGS on finite healthcare dollars, the results of NGS panels can lead to matches with drugs that are yet to be approved in Canada. Additionally, the matched drug may not be publicly reimbursed, putting the onus on the patient to pay for the therapy themselves. In effect, testing in this setting leads to greater uncertainty if the matched drug is not funded and if no suitable clinical trial is available. These circumstances were highlighted by the IMPACT study [29], where only 5% of patients could be placed on targeted drugs based on their tumour genotype. Assessing the cost-effectiveness of molecular testing becomes extremely complicated when there is no assurance that a therapeutic decision can be made on its basis.

Canadian NGS clinical guidelines conclude that “the use of NGS in place of companion diagnostic tests depends on the funding of NGS-based testing across Canada, for which no process is currently defined” [16].

### 1.4. The Way Forward

Uncertainty lies around the evidence base in measuring the health economic impact and has slowed translation from bench to bedside [30]. As noted previously, HTA processes in Canada and elsewhere are not structured to recommend precision oncology technologies without robust comparative data. The uncertainty faced by HTA bodies owing to basket and umbrella trials, often without conventional comparators, has created a need for supplementary real-world evidence (RWE). Mining registries and administrative or clinical databases can potentially generate valuable evidence to inform clinical and regulatory policy. As regulators in many countries have acknowledged, RWE can also drive custom HTA processes in precision oncology and inform funding decisions [1,2,5,11,23,31]. Indeed, Health Canada has published a draft guidance on RWE in oncology [32,33,34]. However, as noted earlier, specific HTA pathways for precision oncology—with or without provisions for RWE—are yet to appear in Canada or elsewhere.

We hypothesize that the current key gap is in the lack of clinical utility data collection to address the health economic uncertainty inherent in single-arm studies. That is, if high-quality clinical utility data can be collected in an efficient and standardized manner, the evidence for molecularly matched therapy will be less ambiguous and will drive wider application in the oncology health ecosystem. To this end, innovations are needed in the gathering of RWE for tests and therapeutics. 

The generation of oncology RWE sufficient to inform HTA policies on precision oncology has two overarching prerequisites: First, interoperable systems that collect clinical outcome data efficiently and comprehensively [5,16,35,36]; and second, principles of high-quality RWE collection that conform to standards required for controlled clinical trials 42 [32], including good research principles as outlined in International Conference of Harmonisation guidelines [37]. As discussed below, countries around the world are struggling with the challenge of improving their frameworks and methodologies to gather RWE. Their experiences may inform Canadian initiatives to optimize the collection and dissemination of clinical utility data. 

### 1.5. Goal of the Paper

Canada is not alone; all countries attempting to provide precision oncology to their citizens face similar challenges. However, each is finding its own solutions. The goal of this paper is to draw on international best practices that could inform Canada’s precision oncology strategy. The focus will be on initiatives designed to generate real-world clinical utility data to support the appropriate use of genomic tests and tumour-agnostic treatments with proven outcome benefits.

## 2. Materials and Methods

### Search Strategy and Selection Criteria

We identified recent or current personalized medicine initiatives worldwide through PubMed, using the following search terms combined logically: incremental benefit, national/federal/global/international, funding, cancer/oncology, biomarkers, big data/crowdsourcing/database, clinical utility/real-world evidence/RWE, NGS/next-generation sequencing, personalized/precision, health/medicine, for review articles published between 1 January 2016 and 1 March 2021. Reference lists of papers were also hand-searched for relevant publications back to 1 December 2005, with an emphasis on oncology. A supplementary Google search was performed for ‘personalized medicine’ and the names of 20 mid- to high-income countries or regions (Australia, Belgium, Brazil, Canada, China, Europe, France, Germany, Hungary, Japan, Korea, The Netherlands, New Zealand, South Africa, Spain, Scandinavia, Switzerland, Taiwan, UK, and USA). A supplementary PubMed search was then conducted for publications from named country-specific programs (e.g., *All of Us*). A final list was generated based on the authors’ evaluation of the initiatives’ relevance to the scope of this review.

## 3. Results

Across 37 countries in Europe, Australasia, Africa, and the Americas, 39 initiatives were identified that could lead to RWD on the clinical utility of oncology biomarkers (Supplemental Tables S1 and S2). Of these, six were international in scope, four were federal (European), and the remaining 29 were national. The six international projects ranged from genomic databases/platforms originating from single academic centres (e.g., cBioPortal for Cancer Genomics [38]) to global strategic efforts (e.g., the World Economic Forum Global Precision-Medicine Council [39,40]).

The European projects included governmental plans to harmonize health infrastructure across the EU [5,41], as well as public–private research consortia to standardize protocols and validate biomarkers, such as CANCER-ID [36]. Nationally, several countries were accumulating vast databases of population genomic data, such as *All of Us* in the USA [42], the Korean Genome Project [43], and Genomics England’s 100,000 Genome Project [44,45,46].

While all these initiatives appear to have the potential to yield RWD on the clinical utility of oncology biomarkers eventually, most are at early stages of development or currently focussing on non-oncology targets. For example, Genome Canada’s four-phase precision health strategy is currently in phase 2, focussing on rare disease; cancer will feature in phase 3. The vision of the Global Alliance for Genomic Health (GA4GH) is that, by 2022, genomic and health-related data will be shared responsibly across worldwide ‘learning health systems’ that will allow translation of research findings into clinical application [47]. However, most of the 24 driver projects of GA4GH, which include *All of Us* [42] Australian Genomics [48], and Genomics England [46] are still working on infrastructure, standardization, or data collection.

Nevertheless, we identified several programs that could provide helpful insights for Canada (Table 1 and Table 2), as discussed below.

In order to make precision oncology a reality for Canadian patients, the value of routine access to publicly funded NGS testing and molecularly matched therapies must be demonstrated with acceptable evidence generated both in the real world and in controlled clinical studies. We highlighted four examples of international work that could inspire and inform the Canadian precision oncology effort: Genomic Medicine France 2025, UNICANCER, the German Medical Informatics Initiative, and CANCER-ID. These examples demonstrated different approaches to achieving interoperability of data systems while protecting patient privacy, and overcoming jurisdictional silos and public–private conflicts of interest and funding. Similarities and differences between key challenges associated with the four initiatives are displayed in Table 2.

**Figure 1:**
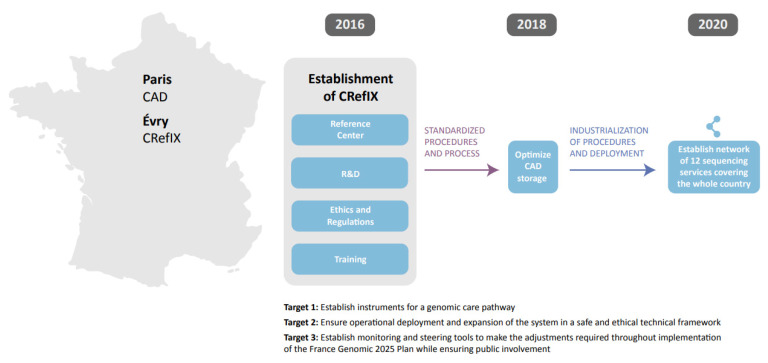
The Genomic Medicine France 2025 initiative provides one model for nationwide sharing of cancer genomic data. Shown here are two hubs where data are generated and disseminated. The Collecteur analyseur de données (CAD) in Paris is a National Center for Intensive Calculation, capable of processing and analyzing large volumes of data and providing primary services for professional healthcare providers in the framework of their care pathways (in silico tests, and aids to decision-making in diagnosis, establishing prognosis, and designing therapeutic strategies). The town of Évry has established a Centre de Référence, d’Innovation, d’eXpertise et de transfert (CRefIX) through public–private partnerships with key national players. Other such hubs can be established using the process outlined here. R&D, Research and Development.

In 2007, in response to the arrival of molecular targets for novel therapeutics in oncology, the French National Cancer Institute set up a national network of 28 hospital Molecular Genetics Centers for Cancer (MGCCs). In 2015, the MGCCs received funding to develop NGS technology, and all private and public laboratories in France were then funded to provide molecular testing in oncology. Genomic Medicine France 2025 is a strategic plan implemented in 2018 to “introduce more systematic sequencing into the health care pathway and oncology practice” [49] (Figure 1).

Genomic Medicine France 2025 identified four major challenges: the challenge of making genomic medicine available to all patients; the scientific and clinical challenge of matching molecular results to therapeutic benefit; the technological challenge of converging and interpreting ‘big data’ from disparate sources; and lastly, the economic challenges of health expenditure versus the opportunity to develop new infrastructure. The French plan aims to: position France as a leader in the field; set up a ‘generic care pathway’ for genomic medicine available to all affected patients; and, set up a framework to drive innovation, capitalization, and economic growth in the field. Four pilot projects were established to assess the effectiveness of the approach and the feasibility of implementing high-throughput screening across the healthcare system [50].

The French genomics effort is remarkable for several reasons: its national scope; the coordinated effort and support from a public funding agency; the uniform testing strategies; and the quality of the science emerging on implementation.

Canada does not have a national biomarker strategy or uniform testing approaches. Healthcare is organized at the provincial level, so few healthcare initiatives have national scope. We believe that one of the strengths of Genomic Medicine France 2025 is that it distills many complex issues into four, clear challenges that encompass the elements that need to come together to make genomic medicine available to all patients who would benefit. Canada has the same need for integration of healthcare, research, and economics as identified by France, so it would benefit from a similar approach, with the obstacles clearly defined and multiple activities in genomic medicine organized under one large program across the country.

**Figure 2:**
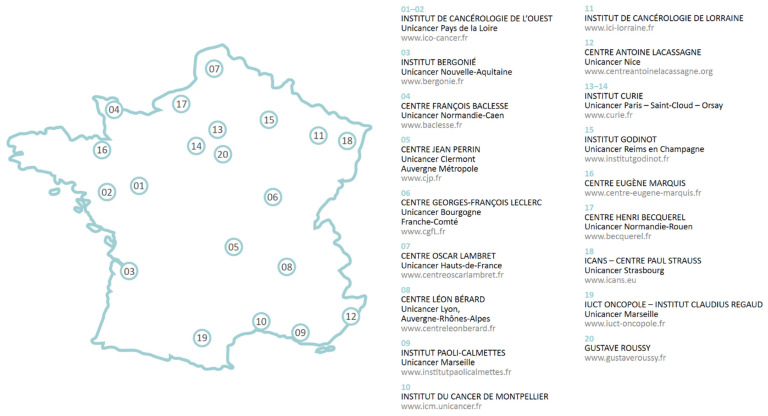
UNICANCER, another French initiative, structured its network of Comprehensive Cancer Centers around seven inter-regions corresponding to a set of regional research coordination centres, or ‘canceropoles’. These include: Grand Ouest (West), Grand Sud Ouest (South-West), Île-de-France (Paris and suburbs), Nord Ouest (North-West), LARA (Lyon, Auvergne, Rhône-Alpes), and PACA (South-East areas). The canceropoles work in association at the territorial level with teams from state-funded scientific organizations (including the Institut National de la Santé et de la Recherche Médicale, the Centre National de la Recherche Scientifique, and universities), university teaching hospitals, FCCCs, the pharmaceutical industry, and players in biotechnology, with the aim of enhancing cooperation and data sharing. FCCC, French Comprehensive Cancer Center.

Additionally, in France and on a smaller scale, another helpful case study for Canada is that of UNICANCER [51], an alliance of 18 FCCCs across the country (Figure 2). These private, non-profit hospitals, financed by the French national health plan, have an integrated approach to patient care, teaching, and research. UNICANCER claims to be the leading academic promoter of oncology clinical trials in Europe [51].

Currently, Canadian provinces have differing data sharing and transfer rules, and most hospital systems do not share data, even within a single city; certainly, data are not shared between public and private entities. The example of UNICANCER is encouraging in that it demonstrates the benefits of navigating around barriers, to harness the collective power of clinical and genomic data in cancer for patient care. By contrast, in the USA, health maintenance organizations can force network institutions to use the same electronic health platform and patients to sign over their healthcare data for research purposes in order to gain entry into their network. Such is the case of Flatiron Health, Inc., which has developed a highly efficient data analytics platform to obtain RWD. Flatiron data have already been utilized in several comparative efficacy analyses for these rare molecularly defined forms [52].

Given that healthcare is a provincial responsibility, Canada will likely never have a single national electronic health platform. Adapting the Flatiron model to the Canadian legal framework will require ownership of healthcare data to be retained by the patients. Consequently, moving data from one provincial jurisdiction to another is fraught with difficulties. A federated model becomes necessary, wherein data are analyzed together across jurisdictions through technology platforms, but never leaves the institution or province. In these models, data are harmonized within the individual institutions and then analyzed in a secure manner across institutions. Such a model, built upon a single platform, is being pursued in pilot form by the Canadian Personalized Health Information Network [53]. Canadian federal government leadership would be beneficial because this approach will depend on efforts by many organizations, including open collaboration between provincial healthcare systems and industry. National standards would also be required, such as the use of mCODE for clinical data [54].

**Figure 3:**
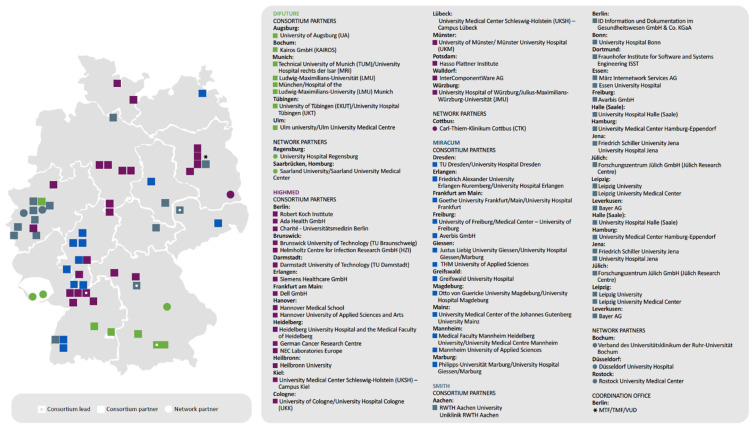
The German Medical Informatics Initiative. The German Federal Ministry of Education and Research provides funding to several consortia within the scope of its Medical Informatics Initiative. Each consortium comprises multiple university hospitals, plus additional partners such as research and higher education institutions, businesses, and non-university hospitals. The consortia work together to create a cross-organizational basis for the exchange of data from research and patient care.

The German Medical Informatics Initiative [55] has started to work on overcoming such data barriers on a national scale (Figure 3). The German Federal Ministry of Education and Research has committed 180 million Euros through 2022 for the program, a joint commitment by Germany’s university hospitals, research institutions, businesses, health insurers, and patient advocacy groups. The intent is “to harness research findings to the direct benefit of patients”, [55] with interoperable data systems that allow for personalized medicine. University hospitals are currently working with relevant partners to develop strategies for shared data use, with the goal of establishing data integration centres and IT solutions.

Canada faces the same challenges as Germany. Even within a single Canadian hospital there are multiple data systems that cannot communicate, which makes sharing and linking data such as mutation information and clinical features—essential for demonstration of clinical utility—unwieldy and time-consuming. Based on the German experience, a Canadian medical informatics initiative would require substantial government funding and buy-in at the earliest stages from university hospitals.

**Figure 4:**
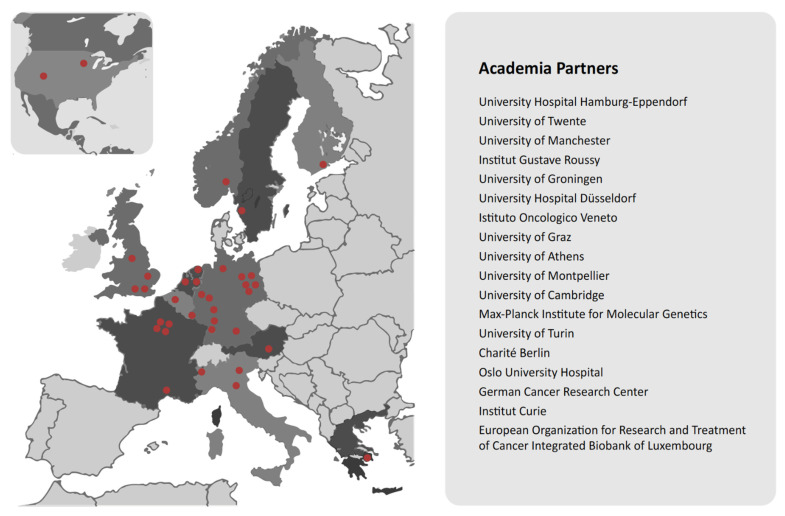
The CANCER-ID Project, operating across multiple countries in Western Europe as well as in the USA, provides a model for sharing of genomic data, specifically for the implementation of liquid biopsies in cancer care. The consortium partners comprise a network of experts in the fields of tumour biology, biomarker development, clinical sciences, and bioinformatics.

CANCER-ID [56] is the final example we would like to propose as an informative model for Canada (Figure 4). CANCER-ID is supported by Europe’s Innovative Medicines Initiative (IMI), which in turn is funded jointly by the EU and the European pharmaceutical industry. IMI has a budget of 3.276 billion euros over 6 years. CANCER-ID is a huge, complex public–private partnership of 36 entities from 13 countries, including the USA, tasked with establishing standard protocols for, and clinical validation of, blood-based biomarkers.

CANCER-ID’s approach overcomes many of the challenges of demonstrating the clinical utility and validity of rare cancer biomarkers, such as small sample sizes and a disconnect between lab results and clinical outcomes. The multi-institutional partnerships of CANCER-ID, which permit shared genomics and clinical data, combined with a data integration platform that allows for natural language processing, and other innovations and integral clinical evaluation, together create a powerful machine for RWE generation that could be emulated by Canada and other jurisdictions.

## 4. Discussion

Globally, many healthcare systems face the same challenges in realizing the potential of precision oncology. The goal of this paper was to draw on international best practices that could inform Canada’s approach, particularly projects designed to generate real-world clinical utility data to support the appropriate use and funding of genomic tests and tumour-agnostic treatments. We have identified 39 initiatives across 37 countries in Europe, Australasia, Africa, and the Americas, which we believed could lead to RWD on the clinical utility of oncology biomarkers, either in the short or long term. These ranged from global strategic plans on personalized medicine to oncology databases originating from single institutions.

It was immediately clear that the vision of seamless integration of standardized genomic and clinical data to provide HTA bodies with real-time data on which to make funding decisions remains just that—a vision. However, many jurisdictions, institutions, and industry bodies are investing substantially in that future. Among the initiatives evaluated, main themes such as the need for collaboration and systems to support data harmonization were apparent.

We highlighted four examples of international work that could inspire and inform the Canadian precision oncology effort: Genomic Medicine France 2025, UNICANCER, the German Medical Informatics Initiative, and CANCER-ID. These examples demonstrated different approaches to achieving interoperability of data systems while protecting patient privacy and overcoming jurisdictional silos and public–private conflicts of interest and funding.

The four shortlisted initiatives have in common a dedicated effort to harmonize data collection and data interpretation across multiple jurisdictions, which is one of the critical elements for success in a country where healthcare is fragmented, with complex oversight. Drawing lessons from these initiatives with respect to the agreements, scope of practice, and methods of data collection and standardization would be an important first step in Canada. The four highlighted projects embody this process, from multiple institutions in one country (UNICANCER) to all institutions within one country (Genomics Medicine in France 2025 and the German Medical Informatics Initiative) to multiple countries (CANCER-ID). The key in all cases appears to be buy-in and commitment from the funding bodies. In Canada, this means complicated discussions that include provincial and territorial ministries are required. Making use of our national platforms, including CADTH and Health Canada, to engage at the provincial and territory levels could offer a useful starting point upon which to build the vision.

The initiatives outlined in the article highlighted the need for collaboration, partnership across multiple jurisdictions, and data harmonization. Generating RWD in precision oncology that is acceptable to clinicians, patients, and Canadian HTA bodies is essential. To do this Canada must bring together government bodies, university hospitals, researchers, data scientists, industry, patients, and others to create a national biomarker strategy, and the infrastructure needed to make precision oncology a reality in a public healthcare system.

## Figures and Tables

**Table 1:** National and Federal Precision Medicine Programs of Interest.

Name	Country	Purpose	Description	Links	Benefits
Genomic Medicine France 2025	France	To prepare for the integration of genomic medicine into the care pathway and management of common diseases with genomic medicine available to all affected patients in the country by 2025.	Development of a 10-year national plan, with a total investment of 670 million euros of which 230 million is from industry and the rest from the French government.	https://solidarites-sante.gouv.fr/IMG/pdf/genomic_medicine_france_2025.pdf (accessed on 21 July 2021)	The savings for the French healthcare system are potentially large and expected to be accompanied by social and economic benefits resulting from a significant improvement in healthy life expectancy.
UNICANCER	France	To build on the historical community of the French Comprehensive Cancer Centers (FCCCs) to facilitate cooperation and creation of synergies between different teams within the network.	UNICANCER groups together with the 20 FCCCs, which are private, non-profit hospitals located throughout the French territory. FCCCs contribute to the public hospital service in accordance with statutory fees, with no additional charge to patients.	http://www.unicancer.fr/ (accessed on 14 June 2021)	UNICANCER helps FCCCs access latest innovations for the benefit of their patients. It also advocates for FCCCs with public authorities and focusses on promoting their organization model in cancer care.
German Medical Informatics Initiative	Germany	To create a framework in which all of Germany’s university hospitals join forces with research institutions, businesses, health insurers, and patient advocacy groups to harness research findings for the direct benefit of patients.	The German Federal Ministry of Education and Research is investing a total of 180 million euros in the Medical Informatics Initiative (MII) through 2022.	https://www.medizininformatik-initiative.de/en (accessed on 18 July 2021)	The MII initiative enables collaboration between medical and IT professionals with researchers and scientists across multiple disciplines in German university hospitals. The shared goal to digitally capture hospital healthcare data and make it available for research is intended to help provide patients with better medical treatments.
CANCER-ID	European Consortium	The CANCER-ID project aims to develop new, less invasive ways of capturing cancer cells and genetic material from tumours using blood samples, and analyzing them for clues as to what treatment is needed and how to apply them.	CANCER-ID is funded by the Innovative Medicines Initiative (IMI), which currently involves 36 partners from 13 countries. Contributions to the project of currently 8.2 million euros by industrial partners are complemented by funding from the IMI Joint Undertaking, resulting in a total budget of 16.7 million euros.	https://www.cancer-id.eu/the-project/ (accessed on 14 July 2021)	CANCER-ID brings together experts from academic and clinical research, innovative small- to medium-sized enterprises, diagnostics companies, and the pharmaceutical industry to help establish the clinical utility of liquid biopsies in cancer care.

**Table 2:** Key challenges identified through the shortlisted initiatives.

	Canadian Challenge		
	Access to Publicly Funded Next-Generation Sequencing Testing	Access to Publicly Funded Molecularly Matched Therapies	Harmonized Real-World Data Collection and Interoperable Real-World Evidence
Genomic Medicine France 2025	X	X	X
UNICANCER		X	X
German Medical Informatics Initiative			X
CANCER-ID	X		X

## Data Availability

All data used, with the exception of two personal communications, are publicly available from the sources cited in Results.

## Supplementary Materials

The following are available online at https://www.mdpi.com/article/10.3390/curroncol29100572/s1, Table S1: International Precision Medicine Initiatives, Table S2: National and Federal Precision Medicine Initiatives.
