# Supplementary material for: Precision Oncology in Canada: Converting Vision to Reality with Lessons from International Programs
Source: Curr Oncol. 2022 Sep 30;29(10):7257–71. doi: 10.3390/curroncol29100572 (PMC9600134; doi:10.3390/curroncol29100572)
Supplement: Supplementary file 1 [file curroncol-29-00572-s001.zip › curroncol-1786377-supplementary.pdf]

Current Oncology MDPI

Supplementary materials for Precision oncology in Canada: Converting vision to reality with lessons from international programs

Supplemental Appendix

Table S1. International Precision Medicine Initiatives
Table S2. National and Federal Precision Medicine Initiatives

Table S1. International Precision Medicine Initiatives.

Name	Purpose	Description	Links
cBioPortal for Cancer Genomics [1]	To explore large-scale cancer genomic data sets that host data from large consortium efforts, as well as publications from individual labs.	The genomics database was first developed at the Memorial Sloan Kettering Cancer Center. It is now a multi-institutional portal.	https://www.cbioportal.org
G2MC: Global Genomic Medicine Collaborative [2]	To catalyze the implementation of genomic tools and knowledge into healthcare delivery globally.	Originated as an action collaborative in the US National Academies of Medicine. It is now an independent charitable organization.	https://g2mc.org/
GA4GH: Global Alliance for Genomic Health	To enable responsible genomic data sharing within a human rights framework.	International, nonprofit alliance of 600+ leading organizations working in healthcare, research, patient advocacy, life science and information technology.	https://www.ga4gh.org/
ICPerMed: International Consortium for Personalized Medicine [3,4,5]	To support a personalized medicine science base through a coordinated approach to research.	40 funding bodies from EU member states and beyond, including Canada. Consortium members include public and private “not-for-profit” health research funding and policy organizations.	https://www.icpermed.eu
The International Cancer Genome Consortium Accelerating Research in Genomic Oncology (ICGC ARGO)	To uniformly analyze specimens from 100,000 donors with high-quality clinical data in order to address outstanding questions that are vital in the quest to defeat cancer.	A data platform that will link genomic data to clinical and health information across the cancer spectrum.	https://platform.icgc-argo.org
World Economic Forum Global Precision Medicine Council [6,7]	To identify and evaluate challenges to the implementation of precision medicine in different settings, countries, cultures and socioeconomic environments.	Council consists of 40 leaders from the public and private sectors, civil society and academia.	https://www.weforum.org/reports/precision-medicine-vision-statement-a-product-of-the-world-economic-forum-global-precision-medicine-council

Table S2. National and Federal Precision Medicine Initiatives.

Name	Country/Region	Purpose	Description	Links
Australian Genomics Health Alliance	Australia	To build the evidence to inform the integration of genomic medicine into mainstream healthcare.	National government-funded research alliance of more than 80 organizations.	https://www.australiangenomics.org.au/
BeMGI: Belgian Medical Genomics Initiative [8]	Belgium	To understand the biology of disease to predict clinical outcomes from genomic information and fulfil a pilot role toward concerted integration of genomic information in clinical care in Belgium.	Federal government-funded alliance of academic hospitals.	https://www.belspo.be/belspo/fedra/proj.asp?l=en&COD=P7%2F43
Genomas Brasil: Brazilian National Program of Genomic and Precision Health	Brazil	To establish the reference genome of the Brazilian population and institute a national database of genomic and clinical data.	National technology and innovation program.	https://www.progressa.com.br/post/genomas-brasil-the-brazilian-national-program-of-genomic-and-precision-medicine
CanDIG [9]	Canada	To allow researchers at participating sites to analyze national scale genomic data while keeping data private and under local control.	National pan-hospital clinical research network.	https://www.distributedgenomics.ca/
CIHR Personalized Medicine Signature Initiative	Canada	To develop an evidence base on assessment and where appropriate, implementation of innovative diagnostics (laboratory diagnostics and medical imaging) into health policy and practice; to stimulate the discovery, validation, and translation of biomarkers, targets and genomic signatures for risk prevention; and to foster the development and validation of diagnostics based on these biomarkers, targets and genomic signatures.	In collaboration with federal and provincial health authorities, multiple funding parties, clinicians, researchers and industry.	https://cihr-irsc.gc.ca/e/43707.html
CPHIN: Canadian Personalized Health Information Network [10]	Canada	To use technology and data to improve health outcomes for Canadians.	Not-for-profit that partners with entities in both the public and private sectors.	www.cphin.ca
Genome Canada Precision Health Strategy [11]	Canada	To develop and apply genomic-based technologies for the economic and social benefit of Canadians.	Not-for-profit funded by the Government of Canada.	https://www.genomecanada.ca/en/about
Oncology Outcomes	Canada	To serve as a highly experienced resource for oncology outcomes and real-world queries from research collaborators and industry partners in order to ensure the quality and integrity of results, and to	RWE-based research.	https://oncoutcomes.com/

		accelerate the transformation of projects into clinical practice and policy.		
P3G2: Public Population Project in Genomics	Canada	To provide expert guidance, training and assistance in the development of policy documents to support researchers in managing important ethical aspects of their projects.	Expert guidance.	http://p3g2.org
Estonia Program for Personal Medicine	Estonia	To implement personalized medicine to impact disease prediction, prevention and treatment on a national scale.	Federal infrastructure initiative.	https://estonia.ee/getting-personal-with-personalised-medicine/
B1MG: Beyond 1 Million Genomes project	Europe	To create a network of genetic and clinical data across Europe.	A project involving 23 European countries.	https://b1mg-project.eu/ ; https://elixir-europe.org/about-us/who-we-are
CANCER-ID [12]	Europe	To establish standard protocols for clinical validation of blood-based biomarkers.	Public-private consortium supported by Europe's Innovative Medicines Initiative (~40 partners from 13 countries).	https://www.cancer-id.eu/the-project/
EHDS: European Health Data Space [13,14]	Europe	To promote better exchange and access to different types of health data (electronic health records, genomics data, data from patient registries, etc.), not only to support healthcare delivery, but also for health research and health policy.	European commission in collaboration with member states.	https://ec.europa.eu/health/ehealth/dataspace_en
European Alliance for Personalized Medicine [13,15]	Europe	To improve patient care by accelerating the development, delivery and uptake of personalized medicine and earlier diagnostics through consensus.	A multi-stakeholder alliance of patient, scientific and commercial organizations.	https://www.euapm.eu
Finnish personalized medicine project	Finland	To help in implementing the health sector growth strategy and strengthen national research, development and innovation activities.	Government-funded alliance of various academic centres.	https://stm.fi/en/personalized-medicine
ESME: Epidemiological Strategy and Medical Economics program [16]	France	To centralize existing real-world patient data in oncology.	Network of 100 international and domestic multidisciplinary experts	http://www.unicancer.fr/en/esme
Genomic Medicine France 2025 [17,18,19,20]	France	To prepare for the integration of genomic medicine into the care pathway and management of common diseases with genomic medicine available to all affected patients in the country by 2025.	Multidisciplinary national plan.	https://solidarites-sante.gouv.fr/IMG/pdf/genomic_medicine_france_2025.pdf
HealthdataHub France	France	To provide easy, unified, transparent and secure access to health data for researchers, entrepreneurs and study managers to improve quality of care and support for patients.	Nationwide data platform.	https://www.health-data-hub.fr
UNICANCER	France	To build on the historical community of the FCCCs to accentuate the cooperation and creation of	Government-funded alliance within the FCCC network.	http://www.unicancer.fr/en/unicancer-group/organisation

		synergies between different teams within the network.		
German Medical Informatics initiative [21]	Germany	To create a framework in which all of Germany's university hospitals join forces with research institutions, businesses, health insurers and patient advocacy groups to harness research findings for the direct benefit of patients.	Governmental informatics initiative.	https://www.medizininformatik-initiative.de/en/about-initiative
MASTER: Molecularly Aided Stratification of Tumor Eradication Research program [22]	Germany	To harness the clinical potential of full genetic analyses and individually tailored therapy in adults with advanced cancers and patients with rare tumours.	Innovation driver for personalized oncology.	https://www.icpermed.eu/en/master-innovation-driver-for-personalised-oncology-769.php
Japan CGM (Cancer Genomic Medicine) program [23,24]	Japan	To explore the implementation of cancer genomic medicine (CGM) in Japan.	National health insurance system.	https://www.ncbi.nlm.nih.gov/pmc/articles/PMC7443380/
Korea precision medicine project [25]	Korea	To explore the field of genomic medicine in Korea and the policies/regulations required to minimize risks.	Expert guidance.	https://www.oecd-ilibrary.org/sites/3b1ee34f-en/index.html?itemId=/content/component/3b1ee34f-en
South African Precision Medicine Program	South Africa	To contribute to a more proactive and preventive approach to health by expanding research to cover genome-wide investigations pertaining to the burden of disease in Southern Africa and to assess the impact of genomic variants on the health of the indigenous populations of Africa.	Extramural research.	https://www.samrc.ac.za/extramural-research-units/precision-and-genomic-medicine
Swiss SPHN Initiative [26,27,28]	Switzerland	To contribute to the development, implementation and validation of coordinated data infrastructures in order to make health-relevant data interoperable and shareable for research in Switzerland.	Federative approach to building upon and supporting existing data sources and infrastructures across the country.	https://www.samw.ch/en/Projects/Overview-of-projects/Personalized-Health/SPHN.html
Taiwan National Precision Medicine Initiative [29]	Taiwan	To achieve tailored medical treatment and health management for everyone in Taiwan.	Consortium of Academia Sinica and 15 medical centre-level hospitals in Taiwan.	https://tpmi.ibms.sinica.edu.tw/www/en/precision-medicine/
Genomics England 100,000 Genome Project [30,31,32]	UK	To support the National Health Service (NHS) in delivering genomic testing for patients as part of routine healthcare, and provide health data and technology needed to make new discoveries and create more effective, targeted medicines.	Genomics England and the UK's NHS' collaboration.	https://www.genomicsengland.co.uk/about-genomics-england/the-100000-genomes-project/

UK Genomic Medicine Service [31,32,33]	UK	To develop a network of seven Genomic Laboratory hubs to deliver genomic tests and collect a wide range of data in the National Test Directory.	National genomic medicine service.	https://publications.parliament.uk/pa/cm201719/cmselect/cmsctech/349/34907.htm
Cancer Moonshot [34]	US	To accelerate scientific discovery in cancer, foster greater collaboration and improve the sharing of data.	A large community of investigators and clinicians dedicated to expediting cancer research.	https://www.cancer.gov/research/key-initiatives/moonshot-cancer-initiative
CSER2: Clinical sequencing evidence-generating research [35]	US	To rapidly advance the knowledge necessary to develop best practices for implementing genomic sequence data into clinical care.	National multi-site research consortium.	https://www.genome.gov/Funded-Programs-Projects/Clinical-Sequencing-Evidence-Generating-Research-CSER2
IGNITE: Implementing GeNomics In practice [30,36,37]	US	To enhance the use of genomic medicine by supporting the development of methods for incorporating genomic information into clinical care, and exploration of the methods for effective implementation, diffusion and sustainability in diverse clinical settings.	NIH funded network of six projects and a coordinating centre.	https://www.genome.gov/Funded-Programs-Projects/Implementing-Genomics-in-Practice-IGNITE
Informatics Technology for Cancer Research (ITCR) program [38]	US	To support a wide range of informatics tools to serve current and emerging needs across the cancer research continuum.	NCI-funded investigator-initiated, research-driven informatics technology development spanning all aspects of cancer research.	https://itcr.cancer.gov
OncoKB	US	To provide a curated resource on genomic alterations in cancer, including biological and clinical information. This tool assesses the level of evidence and assigns an actionability score to individual mutational events for clinical decision making.	Oncology database developed at Memorial Sloan Kettering Cancer Center	https://www.oncokb.org
US Precision Medicine Initiative (All of Us Research Program)	US	To uncover paths toward delivering precision medicine by taking into account individual differences in genes, environment and lifestyle.	Federal governmental funded, high-quality, curated genomic database.	https://allofus.nih.gov/ https://acd.od.nih.gov/working-groups/pmi.html

References

1. Zhang R, Li Q, Fu J, et al. Comprehensive analysis of genomic mutation signature and tumor mutation burden for prognosis of intrahepatic cholangiocarcinoma. *BMC Cancer.* 2021;21(1):112.
2. Ginsburg GS. A Global Collaborative to Advance Genomic Medicine. *Am J Hum Genet.*2019; 104(3):407-409.
3. Vicente AM, Ballensiefen W, Jonsson JI. How personalised medicine will transform healthcare by 2030: the ICPerMed vision. *J Transl Med.* 2020;18(1):180.
4. Aaviksoo A. ICPerMed International Consortium. Action plan: Actionable research and support. https://www.icpermed.eu/media/content/ICPerMed_Actionplan_2017_web.pdf, accessed Feb 26, 2021.
5. Venne J, Busshoff U, Poschadel S, et al. International consortium for personalized medicine: An international survey about the future of personalized medicine. *Per Med.* 2020;17(2):89-100.
6. Precision Medicine Vision Statement: A product of the World Economic Forum Global Precision Medicine Council. https://www.weforum.org/reports/precision-medicine-vision-statement-a-product-of-the-world-economic-forum-global-precision-medicine-council, accessed March 2, 2021.
7. Dana G. Why precision medicine won't transform healthcare – but governance could. https://www.weforum.org/agenda/2020/05/why-precision-medicine-won-t-transform-healthcare-but-governance-could/, accessed March 1, 2021.
8. Belgian Medical Genomics Initiative (BeMGI). https://www.belspo.be/belspo/fedra/proj.asp?l=en&COD=P7%2F43, accessed March 2, 2021.
9. CanDIG. National, distributed, analysis of locally-controlled private genomic data. https://www.distributedgenomics.ca/, accessed Feb 28, 2021.
10. CPHIN: Canadian Personalized Health Information Network. https://www.cphin.ca, accessed March 5, 2021.
11. Genome Canada. A Precision Health Strategy for Canada: Think big, start small , accessed Feb 28, 2021.
12. Weber S, Spiegl B, Perakis SO, et al. Technical Evaluation of Commercial Mutation Analysis Platforms and Reference Materials for Liquid Biopsy Profiling. *Cancers (Basel).* 2020;12(6):1588.
13. Horgan D, Ciliberto G, Conte P, et al. Bringing Onco-Innovation to Europe's Healthcare Systems: The Potential of Biomarker Testing, Real World Evidence, Tumour Agnostic Therapies to Empower Personalised Medicine. *Cancers (Basel).* 2021;13(3).
14. Vayena E. Value from health data: European opportunity to catalyse progress in digital health. *The Lancet.* 2021;397(10275):652-653.
15. European Alliance for Personalised Medicine. https://www.euapm.eu, accessed Feb 28, 2021.
16. Cabel L, Carton M, Pistilli B, et al. Outcome beyond third-line chemotherapy for metastatic triple-negative breast cancer in the French ESME program. *Breast.* 2021;56:18-25.
17. Hofman P, Rouleau E, Sabourin JC, et al. Predictive molecular pathology in non-small cell lung cancer in France: The past, the present and the perspectives. *Cancer Cytopathol.* 2020;128(9):601-610.
18. Group FGMWS, Auzanneau C, Bacq D, et al. Feasibility of high-throughput sequencing in clinical routine cancer care: lessons from the cancer pilot project of the France Genomic Medicine 2025 plan. *ESMO Open.* 2020;5(4).
19. Aviesan. Genomic Medicine France 2025. https://solidarites-sante.gouv.fr/IMG/pdf/genomic_medicine_france_2025.pdf, accessed February 28, 2021.
20. Reda M, Richard C, Bertaut A, et al. Implementation and use of whole exome sequencing for metastatic solid cancer. *EBioMedicine.* 2020;51:102624.
21. Medical Informatics Initiative Germany. About the Initiative. https://www.medizininformatik-initiative.de/en/about-initiative, accessed March 9, 2021.

22. ICPerMed International Consortium. MASTER – Innovation Driver for Personalised Oncology. https://www.icpermed.eu/en/master-innovation-driver-for-personalised-oncology-769.php, accessed Feb 28, 2021.
23. Mukai Y, Ueno H. Establishment and implementation of cancer genomic medicine in Japan. *Cancer Sci.* 2021;112(3):970-977.
24. Mano H. Cancer genomic medicine in Japan. *Proc Jpn Acad.* 2020;96:316–321.
25. Organisation for Economic Co-operation and Development (OECD). Policy issues for the development and use of biomarkers in health. https://www.oecd.org/health/biotech/49023036.pdf, accessed Mar 1, 2021. 2011.
26. Blasimme A, Fadda M, Schneider M, Vayena E. Data Sharing For Precision Medicine: Policy Lessons And Future Directions. *Health Aff (Millwood).* 2018;37(5):702-709.
27. Swiss Personalized Health Network; Report from the National Steering Board 2016-2019. https://zenodo.org/record/4044123#.YbyO1mhOmUk, accessed March 3, 2021.
28. Lawrence AK, Selter L, Frey U. SPHN - The Swiss Personalized Health Network Initiative. *Stud Health Technol Inform.* 2020;270:1156-1160.
29. Taiwan National Precision Medicine Initiative. https://tpmi.ibms.sinica.edu.tw/www/en/precision-medicine/, accessed March 5, 2021.
30. Ginsburg GS, Phillips KA. Precision Medicine: From Science To Value. *Health Aff (Millwood).* 2018;37(5):694-701.
31. McDermott FD, Newton K, Beggs AD, Clark SK. Implications for the colorectal surgeon following the 100 000 Genomes Project. *Colorectal Dis.* 2021;23(5):1049-1058.
32. Turnbull C. Introducing whole-genome sequencing into routine cancer care: the Genomics England 100 000 Genomes Project. *Ann Oncol.* 2018;29(4):784-787.
33. Snape K, Wedderburn S, Barwell J. The new genomic medicine service and implications for patients. *Clin Med (Lond).* 2019;19(4):273–277.
34. Agus DB, Jaffee EM, Van Dang C. Cancer Moonshot 2.0. *Lancet Oncology.* 2021;22(2):164-165.
35. Pfizer Canada ULC. Xalkori (Crizotinib Capsules) Product Monograph, February 3, 2021.
36. Duong BQ, Arwood MJ, Hicks JK, et al. Development of customizable implementation guides to support clinical adoption of pharmacogenomics: Experiences of the Implementing GeNomics In pracTicE (IGNITE) Network. *Pharmgenomics Pers Med.* 2020;13:217-226.
37. Hicks JK, El Rouby N, Ong HH, et al. opportunity for genotype-guided prescribing among adult patients in 11 US health systems. *Clin Pharmacol Ther.* 2021;110(1):179-188.
38. Warner J, Klemm J. Informatics tools for cancer research and care, bridging the gap between innovation and implementation. *JCO Clinical Cancer Informatics* 2020; 4:784-786
